# Supplementary material for: In Silico and In Vitro Study towards the Rational Design of 4,4′-Disarylbisthiazoles as a Selective α-Synucleinopathy Biomarker
Source: Int J Mol Sci. 2023 Nov 17;24(22):16445. doi: 10.3390/ijms242216445 (PMC10671360; doi:10.3390/ijms242216445)
Supplement: Supplementary file 1 [file ijms-24-16445-s001.zip › ijms-2695625-supplementary.pdf]

In Silico and In Vitro Study towards the Rational Design of 4,4′-Disarylbisthiazoles as a Selective α-Synucleinopathy Biomarker

Bright C. Uzuegbunam [1], Junhao Li [2], Wojciech Paslawski [3], Wolfgang Weber [1], Per Svenningsson [3], Hans Ågren [2] and Behrooz Hooshyar Yousefi [4,*]

[1] Department of Nuclear Medicine, Technical University of Munich, 81675 Munich, Germany
[2] Department of Physics and Astronomy, Uppsala University, 751 20, Uppsala, Sweden
[3] Department of Clinical Neuroscience, Karolinska Institute, 171 76, Stockholm, Sweden
[4] Department of Nuclear Medicine, Philipps University of Marburg, 35043 Marburg, Germany
* Correspondence: b.h.yousefi@uni-marburg.de; Tel.: +49-6421-58-65806; Fax: +49-6421-58-62899

Table of Contents

Characterization of the DABTAs………..……………………….……………………………………………....3

^{1}H and ^{13}C spectra of the 4-aryl/heteroarylthiazole-2-carbothioamide intermediate, b……………………...………..3

^{1}H and ^{13}C spectra of the asymmetric DABTAs, d……………………………………...………………………4

^{1}H and ^{13}C spectra of the fluoroethylated and fluoro- and iodoPEGylated DABTAs ………………………..…....6

Competition binding assays with [^{3}H]DVJ (graphs)…………………………………………….……………….....9

Competition binding assays with [^{3}H]PIB (graphs).…………………………………………….…………....…12

In silico studies……………………………………………………………………………………………..16

1. Characterization of the DABTAs

1.1. ^{1}H and ^{13}C spectra of the 4-aryl/heteroarylthiazole-2-carbothioamide intermediate, b

1.1.1. 4-(benzo[d][1,3]dioxol-5-yl)thiazole-2-carbothioamide, b_1

^{1}H (DMSO-d_6) δ 6.07 (s, 2H), 7.00 (d, J = 5.5 Hz, 1H), 7.61 (dd, J = 4.3, 8.3 Hz, 1H), 7.69 (s, 1H), 8.27 (s, 1H), 9.94 (s, 1H), 10.19 (s, 1H). ^{13}C (DMSO-d_6) δ 39.02, 39.19, 39.35, 39.52, 39.69, 39.85, 40.02, 101.30, 106.77, 108.55, 120.25, 121.60, 127.98, 147.54, 147.86, 155.27, 167.64, 186.46

1.1.2. 4-(3-hydroxy-4-methoxyphenyl)thiazole-2-carbothioamide, b_3

^{1}H (DMSO-d_6) δ 3.82 (s, 3H), 7.00 (d, J = 5.6 Hz, 1H), 7.33 – 7.59 (m, 2H), 8.16 (s, 1H), 9.05 (s, 1H), 9.87 (s, 1H), 10.18 (s, 1H). ^{13}C (DMSO-d_6) δ 55.68, 112.05, 113.71, 117.69, 121.19, 126.74, 146.57, 148.18, 155.85, 167.71, 186.62.

1.1.3. 4-(3-fluoro-4-methoxyphenyl)thiazole-2-carbothioamide, b_4

^{1}H (DMSO-d_6) δ 3.89 (s, 3H), 7.21 – 7.29 (m, 1H), 7.85 (d, J = 8.8 Hz, 1H), 7.98 (d, J = 13.0 Hz, 1H), 8.34 (s, 1H), 9.98 (s, 1H), 10.21 (s, 1H). ^{13}C (DMSO-d6) δ 39.02, 39.19, 39.35, 39.52, 39.69, 39.85, 40.02, 101.30, 106.77, 108.55, 120.25, 121.60, 127.98, 147.54, 147.86, 155.27, 167.64, 186.46.

1.1.4. 4-(6-fluoropyridin-3-yl)thiazole-2-carbothioamide, b_8

^{1}H (DMSO-d_6) δ 7.33 (d, J = 8.5 Hz, 1H), 8.54 (d, J = 3.1 Hz, 1H), 8.61 (d, J = 8.7 Hz, 1H), 8.97 (s, 1H), 10.03 (s, 1H), 10.27 (s, 1H). ^{13}C (DMSO-d_6) δ 109.69, 124.24, 128.13, 128.16, 139.67, 139.74, 145.50, 145.63, 151.45, 161.86, 163.75, 168.62, 186.24.

1.1.5. 4-(2-fluoropyrimidin-4-yl)thiazole-2-carbothioamide, b_{10}

^{1}H (DMSO-d_6) δ 8.65 (q, J = 2.4 Hz, 1H), 9.44 (s, 1H), 10.07 (s, 1H), 10.31 (s, 1H). ^{13}C (DMSO-d_6) δ 125.99, 127.09, 127.13, 148.82, 159.47, 159.57, 161.37, 163.08, 169.47, 186.50.

1.1.6. 4-(5-hydroxypyrimidin-2-yl)thiazole-2-carbothioamide, b_{11}

^{1}H (DMSO-d_6) δ 8.43 (d, J = 2.5 Hz, 2H), 8.50 (d, J = 2.5 Hz, 1H), 9.81 (s, 1H), 10.23 (s, 1H), 10.72 (s, 1H). ^{13}C (DMSO-d_6) δ 128.73, 144.52, 150.99, 151.66, 155.06, 168.74, 186.67.

1.1.7. 4-(3-fluoro-4-hydroxyphenyl)thiazole-2-carbothioamide, b_{12}

^{1}H (DMSO-d_6) δ 7.01 (t, J = 8.7 Hz, 1H), 7.69 (dd, J = 2.1, 8.4 Hz, 1H), 7.91 (dd, J = 2.1, 12.7 Hz, 1H), 8.25 (s, 1H), 9.94 (s, 1H), 10.15 (s, 1H), 10.19 (s, 1H). ^{13}C (DMSO-d_6) δ 114.23, 114.38, 117.92, 117.95, 121.50, 122.62, 122.64, 125.59, 125.64, 145.30, 145.40, 150.25, 152.16, 154.69, 154.71, 167.77, 186.46.

1.1.8. 4-(4-hydroxyphenyl)thiazole-2-carbothioamide, b_{13}

^{1}H (DMSO-d_6) δ 6.79 – 6.87 (m, 2H), 7.88 (dd, *J* = 2.2, 8.7 Hz, 2H), 8.14 (d, *J* = 2.1 Hz, 1H), 9.70 (s, 1H), 9.86 (s, 1H), 10.16 (s, 1H). ^{13}C (DMSO-d_6) δ 115.51, 120.45, 124.91, 127.80, 156.03, 157.94, 167.67, 186.61.

1.1.9. 4-(2,4-dihydroxyphenyl)thiazole-2-carbothioamide, b_{16}

^{1}H (DMSO-d_6) δ 6.32 (dd, *J* = 2.3, 8.6 Hz, 1H), 6.41 (d, *J* = 2.2 Hz, 1H), 8.00 (d, *J* = 8.6 Hz, 1H), 8.14 (s, 1H), 9.56 (d, *J* = 1.4 Hz, 1H), 9.91 (s, 1H), 10.13 (s, 1H), 10.14 (s, 1H). ^{13}C (DMSO-d_6) δ 102.84, 107.08, 111.69, 122.59, 130.19, 153.42, 156.33, 158.64, 166.17, 186.60.

1.2. ^{1}H and ^{13}C spectra of the asymmetric DABTAs, d

1.2.1. 5-(4'-(benzo[d][1,3]dioxol-5-yl)-[2,2'-bithiazol]-4-yl)-2-methoxyphenol, d_1

^{1}H (DMSO-d_6) δ 3.82 (s, 3H), 6.09 (s, 2H), 7.03 (dd, J = 8.3, 10.5 Hz, 2H), 7.43 (dd, J = 22, 8.4 Hz, 1H), 7.48 (d, J = 2.2 Hz, 1H), 7.59 (d, J = 8.0 Hz, 2H), 8.11 (s, 1H), 8.21 (s, 1H), 9.21 (s, 1H). ^{13}C (DMSO-d_6) δ 55.65, 101.37, 106.40, 108.69, 112.35, 113.48, 114.94, 115.36, 117.40, 120.30, 126.42, 127.69, 146.71, 147.58, 147.91, 148.20, 155.15, 155.64, 160.03, 160.31.

1.2.2. 4-(benzo[d][1,3]dioxol-5-yl)-4'-(3-fluoro-4-methoxyphenyl)-2,2'-bithiazole, d_2

^{1}H (DMSO-d_6) δ 3.90 (s, 3H), 6.09 (s, 2H), 7.04 (d, J = 8.0 Hz, 1H), 7.29 (t, J = 8.7 Hz, 1H), 7.56 – 7.61 (m, 2H), 7.80 – 7.89 (m, 2H), 8.23 (s, 1H), 8.30 (s, 1H). ^{13}C (DMSO-d_6) 2-δ 56.11, 101.37, 106.39, 108.68, 113.67, 114.13, 115.57, 116.14, 120.29, 122.65, 126.57, 127.64, 147.43, 147.91, 150.65, 152.58, 154.17, 155.20, 160.05, 160.43.

1.2.3. 5-(4'-([1,3]dioxolo[4,5-b]pyridin-6-yl)-[2,2'-bithiazol]-4-yl)-2-methoxyphenol, d_3

^{1}H (DMSO-d_6) δ 9.26 (s, 1H), 8.29 (d, J = 10.6 Hz, 2H), 8.10 (s, 1H), 7.80 (s, 1H), 7.46 (s, 1H), 7.42 (d, J = 7.4 Hz, 1H), 7.01 (d, J = 8.4 Hz, 1H), 6.21 (s, 2H), 3.81 (s, 3H). ^{13}C (DMSO-d_6)1-δ 55.72, 100.88, 112.30, 112.40, 113.52, 115.23, 116.65, 117.52, 124.52, 126.45, 136.85, 140.54, 146.77, 148.30, 152.64, 155.75, 158.23, 159.79, 160.95.

1.2.4. 6-(4'-(3-fluoro-4-methoxyphenyl)-[2,2'-bithiazol]-4-yl)-[1,3]dioxolo[4,5-b]pyridine, d_4

^{1}H (DMSO-d_6/THF-d_8 4:3 vol.) δ 3.92 (s, 3H), 6.22 (s, 2H), 7.25 – 7.33 (m, 1H), 7.82 – 7.89 (m, 3H), 8.29 – 8.38 (m, 3H). ^{13}C (DMSO-d_6/THF-d_8 4:3 vol.) δ 55.90, 100.84, 112.00, 113.45, 113.61, 113.89, 115.99, 116.43, 122.47, 122.50, 124.52, 126.69, 126.74, 136.96, 140.48, 147.57, 147.65, 150.87, 152.80, 152.85, 154.48, 158.26, 160.27, 160.71.

1.2.5. 4-(benzo[d][1,3]dioxol-5-yl)-4'-(6-fluoropyridin-3-yl)-2,2'-bithiazole, d_6

^{1}H (DMSO-d_6) δ 6.07 – 6.12 (m, 2H), 7.04 (dd, J = 2.8, 7.9 Hz, 1H), 7.34 (d, J = 8.5 Hz, 1H), 7.59 (dd, J = 2.8, 10.5 Hz, 2H), 8.23 – 8.27 (m, 1H), 8.48 – 8.52 (m, 1H), 8.54 – 8.61 (m, 1H), 8.89 (s, 1H). ^{13}C (DMSO-d_6) δ 101.40, 106.40, 108.71, 109.85, 110.15, 115.85, 118.31, 120.32, 127.59, 127.90, 127.94, 139.75, 139.81, 145.23, 145.35, 147.64, 147.93, 151.42, 155.28, 159.80, 161.20, 161.84, 163.73.

1.2.6. 6-(4'-(6-fluoropyridin-3-yl)-[2,2'-bithiazol]-4-yl)-[1,3]dioxolo[4,5-b]pyridine, d_8

^{1}H (DMSO-d_6/THF-d_8 4:3 vol.) δ 6.23 (s, 2H), 7.29 – 7.35 (m, 1H), 7.85 (d, J = 5.9 Hz, 1H), 8.34 – 8.40 (m, 2H), 8.54 (s, 1H), 8.61 (s, 1H), 8.93 (d, J = 3.9 Hz, 1H). ^{13}C (DMSO-d_6/THF-d_8 4:3 vol.) 1-δ 100.85, 109.59, 109.89, 112.02, 116.75, 118.24, 124.46, 127.97, 136.97, 139.55, 139.62, 140.49, 145.27, 145.40, 151.66, 152.93, 158.29, 160.42, 161.07, 162.03, 163.92.

1.2.7. 4-(4'-(2-fluoropyrimidin-5-yl)-[2,2'-bithiazol]-4-yl)-[1,3]dioxolo[4,5-b]pyridine, d_{10}

^{1}H (DMSO-d_6/THF-d_8 4:3 vol.) δ 6.22 (s, 2H), 7.85 (d, J = 6.3 Hz, 1H), 8.38 (d, J = 11.8 Hz, 2H), 8.68 (s, 1H), 9.41 (s, 2H). ^{13}C (DMSO-d_6/THF-d_8 4:3 vol.) δ 100.87, 112.00, 116.97, 119.75, 124.42, 126.46, 137.01, 140.50, 148.63, 153.03, 158.32, 158.82, 158.91, 160.16, 161.18, 161.68, 162.90.

1.2.8. 2-(4'-([1,3]dioxolo[4,5-b]pyridin-6-yl)-[2,2'-bithiazol]-4-yl)pyrimidin-5-ol, d_{11}

^{1}H (DMSO-d_6) δ 6.23 (s, 2H), 7.84 (s, 1H), 8.33 (s, 2H), 8.47 (s, 3H), 10.74 (s, 1H). ^{13}C (DMSO-d_6) δ 100.81, 112.27, 116.90, 122.87, 124.42, 136.83, 140.49, 144.61, 150.96, 151.51, 152.62, 154.94, 158.20, 160.41, 160.84

1.2.9. 2-(4'-(benzo[d][1,3]dioxol-5-yl)-[2,2'-bithiazol]-4-yl)pyrimidin-5-ol, d_{12}

^{1}H (DMSO-d_6) δ 6.09 (s, 2H), 7.04 (d, J = 11.4 Hz, 1H), 7.56 – 7.62 (m, 2H), 8.23 (s, 1H), 8.43 – 8.49 (m, 3H), 10.74 (s, 1H). ^{13}C (DMSO-d_6) 2- δ 101.39, 106.43, 108.71, 115.67, 120.32, 122.65, 127.66, 144.60, 147.61, 147.93, 150.94, 151.54, 154.90, 155.18, 160.27, 160.63.

1.2.10. 4-(4'-([1,3]dioxolo[4,5-b]pyridin-6-yl)-[2,2'-bithiazol]-4-yl)-2-fluorophenol, d_{13}

^{1}H (DMSO-d_6) δ 6.22 (s, 2H), 7.05 (t, J = 8.8 Hz, 1H), 7.68 (dd, J = 2.0, 8.4 Hz, 1H), 7.75 – 7.84 (m, 2H), 8.22 (s, 1H), 8.31 (d, J = 1.9 Hz, 2H), 10.20 (s, 1H). ^{13}C (DMSO-d_6) δ 100.81, 112.22, 113.86, 114.01, 115.59, 116.74, 118.10, 118.13, 122.68, 122.71, 124.42, 125.21, 125.26, 136.80, 140.47, 145.37, 145.47, 150.21, 152.12, 152.61, 154.63, 154.65, 158.17, 160.04, 160.71.

1.2.11. 4-(4'-(benzo[d][1,3]dioxol-5-yl)-[2,2'-bithiazol]-4-yl)phenol, d_{14}

^{1}H (DMSO-d_6) δ 6.09 (s, 2H), 6.87 (d, J = 8.7 Hz, 2H), 7.01 – 7.07 (m, 1H), 7.59 (d, J = 7.8 Hz, 2H), 7.85 (d, J = 8.7 Hz, 2H), 8.10 (s, 1H), 8.22 (s, 1H), 9.74 (s, 1H). ^{13}C (DMSO-d_6) δ 101.39, 106.41, 108.71, 114.17, 115.37, 115.66, 120.30, 124.64, 127.67, 127.71, 147.60, 147.93, 155.15, 155.87, 157.94, 160.06, 160.36, 162.35.

1.2.12. 4-(4'-([1,3]dioxolo[4,5-b]pyridin-6-yl)-[2,2'-bithiazol]-4-yl)phenol, d_{15}

^{1}H (DMSO-d_6) δ 6.22 (s, 2H), 6.87 (dd, *J* = 2.8, 8.6 Hz, 2H), 7.80 – 7.87 (m, 3H), 8.11 (d, *J* = 2.7 Hz, 1H), 8.31 (t, *J* = 3.5 Hz, 2H), 9.76 (s, 1H). ^{13}C (DMSO-d_6) δ 100.84, 112.26, 114.42, 115.70, 116.63, 124.49, 124.62, 127.70, 136.82, 140.51, 152.60, 155.93, 157.98, 158.20, 159.85, 160.95.

1.2.13. 4-(4'-([1,3]dioxolo[4,5-b]pyridin-6-yl)-[2,2'-bithiazol]-4-yl)benzene-1,3-diol, d_{22}

^{1}H (DMSO-d6) δ 6.22 (s, 2H), 6.38 (dd, J = 2.3, 8.5 Hz, 1H), 6.45 (d, J = 2.3 Hz, 1H), 7.82 (d, J = 1.9 Hz, 1H), 7.91 (d, J = 8.5 Hz, 1H), 8.12 (s, 1H), 8.27 – 8.35 (m, 2H), 9.59 (s, 1H), 10.31 (s, 1H). ^{13}C (DMSO-d_6) δ 100.77, 102.91, 107.20, 111.52, 112.21, 116.41, 124.48, 129.77, 136.77, 140.44, 152.56, 152.91, 156.31, 158.13, 158.18, 158.63, 161.00.

1.3. ^{1}H and ^{13}C spectra of the fluoroethylated and fluoro- and iodoPEGylated DABTAs

1.3.1. 6-(4'-(5-(2-fluoroethoxy)pyrimidin-2-yl)-[2,2'-bithiazol]-4-yl)-[1,3]dioxolo[4,5-b]pyridine, f_4

^{1}H (DMSO-d_6) δ 4.52 (d, J = 29.9 Hz, 2H), 4.82 (d, J = 48.5 Hz, 2H), 6.23 (s, 2H), 7.81 – 7.90 (m, 1H), 8.31 – 8.37 (m, 2H), 8.48 – 8.59 (m, 1H), 8.67 – 8.78 (m, 2H). ^{13}C (DMSO-d_6) δ 68.05, 68.20, 81.34, 82.67, 100.81, 112.26, 116.97, 123.84, 124.40, 136.83, 140.48, 144.37, 151.44, 152.65, 152.86, 154.53, 158.20, 160.60, 160.75.

1.3.2. 4-(benzo[d][1,3]dioxol-5-yl)-4'-(5-(2-fluoroethoxy)pyrimidin-2-yl)-2,2'-bithiazole, f_5

^{1}H (DMSO-d6) δ 4.48 (t, J = 3.6 Hz, 1H), 4.54 (t, J = 3.5 Hz, 1H), 4.77 (t, J = 5.0 Hz, 1H), 4.87 (t, J = 3.3 Hz, 1H), 6.09 (s, 2H), 7.04 (dd, J = 3.2, 8.2 Hz, 1H), 7.59 (dd, J = 3.1, 8.3 Hz, 2H), 8.24 (d, J = 3.3 Hz, 1H), 8.54 (s, 1H), 8.71 (s, 2H). ^{13}C (DMSO-d_6) δ 68.05, 68.20, 81.35, 82.67, 101.39, 106.43, 108.70, 115.73, 120.33, 123.61, 127.64, 144.35, 147.62, 147.92, 151.43, 152.89, 154.50, 155.21, 160.18, 160.82.

1.3.3. 4-(benzo[d][1,3]dioxol-5-yl)-4'-(5-(2-(2-fluoroethoxy)ethoxy)pyrimidin-2-yl)-2,2'-bithiazole, f_6

^{1}H (DMSO-d6) δ 3.71 (t, J = 3.7 Hz, 1H), 3.75 – 3.80 (m, 1H), 3.82 – 3.87 (m, 2H), 4.34 – 4.40 (m, 2H), 4.49 – 4.54 (m, 1H), 4.61 (t, J = 3.6 Hz, 1H), 6.10 (s, 2H), 7.04 (d, J = 15.6 Hz, 1H), 7.56 – 7.61 (m, 2H), 8.24 (s, 1H), 8.53 (s, 1H), 8.70

(s, 2H). ^{13}C (DMSO-d_6) δ 68.23, 68.81, 69.76, 69.91, 82.41, 83.72, 101.40, 106.43, 108.72, 115.74, 120.34, 123.48, 127.65, 144.30, 147.63, 147.93, 151.71, 152.68, 154.57, 155.22, 160.20, 160.80.

1.3.4. 4-(benzo[d][1,3]dioxol-5-yl)-4'-(5-(2-(2-(2-fluoroethoxy)ethoxy)ethoxy)pyrimidin-2-yl)-2,2'-bithiazole, f_7

^{1}H (DMSO-d_6) δ 3.56 – 3.67 (m, 5H), 3.67 – 3.71 (m, 1H), 3.79 – 3.84 (m, 2H), 4.33 – 4.38 (m, 2H), 4.44 – 4.50 (m, 1H), 4.54 – 4.59 (m, 1H), 6.10 (s, 2H), 7.01 – 7.07 (m, 1H), 7.59 (d, J = 7.7 Hz, 2H), 8.24 (s, 1H), 8.53 (s, 1H), 8.69 (s, 2H). ^{13}C (DMSO-d_6) δ 30.75, 68.25, 68.82, 69.66, 69.81, 69.85, 69.95, 82.43, 83.75, 101.41, 106.44, 108.72, 115.74, 120.33, 123.46, 127.65, 144.28, 147.63, 147.94, 151.73, 152.65, 154.58, 155.21, 160.21, 160.79.

1.3.5. 6-(4'-(5-(2-(2-fluoroethoxy)ethoxy)pyrimidin-2-yl)-[2,2'-bithiazol]-4-yl)-[1,3]dioxolo[4,5-b]pyridine, f_8

^{1}H (DMSO-d_6) δ 3.69 – 3.74 (m, 1H), 3.75 – 3.80 (m, 1H), 3.82 – 3.88 (m, 2H), 4.35 – 4.41 (m, 2H), 4.49 – 4.54 (m, 1H), 4.58 – 4.64 (m, 1H), 6.23 (s, 2H), 7.85 (d, J = 1.9 Hz, 1H), 8.31 – 8.37 (m, 2H), 8.56 (s, 1H), 8.70 (s, 2H). ^{13}C (DMSO-d_6) δ 68.24, 68.82, 69.76, 69.91, 82.42, 83.74, 100.84, 112.29, 116.99, 123.73, 124.43, 136.85, 140.51, 144.32, 151.74, 152.66, 154.61, 158.22, 160.59, 160.79.

1.3.6. 6-(4'-(3-(2-(2-fluoroethoxy)ethoxy)-4-methoxyphenyl)-[2,2'-bithiazol]-4-yl)-[1,3]dioxolo[4,5-b]pyridine, f_9

^{1}H (DMSO-d_6) δ 3.70 – 3.76 (m, 1H), 3.76 – 3.86 (m, 6H), 4.18 – 4.24 (m, 2H), 4.50 – 4.55 (m, 1H), 4.59 – 4.65 (m, 1H), 6.22 (s, 2H), 7.08 (d, J = 8.4 Hz, 1H), 7.57 – 7.64 (m, 2H), 7.82 (d, J = 1.9 Hz, 1H), 8.27 (s, 1H), 8.32 (d, J = 1.5 Hz, 2H). ^{13}C (DMSO-d_6) δ 55.60, 68.04, 69.05, 69.78, 69.93, 82.49, 83.80, 100.81, 111.19, 112.18, 112.22, 115.51, 116.66, 119.32, 124.44, 126.24, 136.80, 140.48, 148.10, 149.52, 152.59, 155.60, 158.17, 159.90, 160.80.

1.3.7. 6-(4'-(3-fluoro-4-(2-(2-(2-fluoroethoxy)ethoxy)ethoxy)phenyl)-[2,2'-bithiazol]-4-yl)-[1,3]dioxolo[4,5-b]pyridine, f_{10}

^{1}H (DMSO-d_6) δ 3.71 (t, J = 3.7 Hz, 1H), 3.75 – 3.80 (m, 1H), 3.82 – 3.87 (m, 2H), 4.34 – 4.40 (m, 2H), 4.49 – 4.54 (m, 1H), 4.61 (t, J = 3.6 Hz, 1H), 6.10 (s, 2H), 7.04 (d, J = 15.6 Hz, 1H), 7.56 – 7.61 (m, 2H), 8.24 (s, 1H), 8.53 (s, 1H), 8.70 (s, 2H). ^{13}C (DMSO-d_6) δ 68.23, 68.81, 69.76, 69.91, 82.41, 83.72, 101.40, 106.43, 108.72, 115.74, 120.34, 123.48, 127.65, 144.30, 147.63, 147.93, 151.71, 152.68, 154.57, 155.22, 160.20, 160.80.

1.3.8. 4-(benzo[d][1,3]dioxol-5-yl)-4'-(4-(2-fluoroethoxy)phenyl)-2,2'-bithiazole, f_{11}

^{1}H (DMSO-d_6) δ 4.24 – 4.29 (m, 1H), 4.30 – 4.35 (m, 1H), 4.70 – 4.75 (m, 1H), 4.80 – 4.85 (m, 1H), 6.09 (s, 2H), 7.04 (d, J = 7.9 Hz, 1H), 7.09 (d, J = 8.9 Hz, 2H), 7.59 (d, J = 8.2 Hz, 2H), 7.97 (d, J = 8.8 Hz, 2H), 8.22 (d, J = 4.5 Hz, 2H). ^{13}C (DMSO-d_6) δ 67.09, 67.24, 81.53, 82.86, 101.40, 106.41, 108.71, 114.87, 115.17, 115.46, 120.31, 126.48, 127.65, 127.70, 147.60, 147.93, 155.18, 155.30, 158.46, 160.27.

1.3.9. 6-(4'-(4-(2-(2-(2-fluoroethoxy)ethoxy)ethoxy)phenyl)-[2,2'-bithiazol]-4-yl)-[1,3]dioxolo[4,5-b]pyridine, f_{12}

^{1}H (DMSO-d6) δ 3.56 – 3.61 (m, 2H), 3.61 – 3.66 (m, 3H), 3.66 – 3.72 (m, 1H), 3.75 – 3.81 (m, 2H), 4.13 – 4.18 (m, 2H), 4.45 – 4.50 (m, 1H), 4.54 – 4.60 (m, 1H), 6.23 (s, 2H), 7.03 – 7.10 (m, 2H), 7.83 (d, J = 1.8 Hz, 1H), 7.92 – 7.99 (m, 2H), 8.22 (s, 1H), 8.32 (d, J = 2.4 Hz, 2H). ^{13}C (DMSO-d_6) δ 67.24, 68.97, 69.67, 69.82, 69.87, 69.97, 82.43, 83.75, 100.82, 112.24, 114.83, 115.25, 116.69, 124.45, 126.13, 127.61, 136.81, 140.49, 152.62, 155.43, 158.18, 158.82, 160.00, 160.85.

1.3.10. 6-(4'-(5-(2-(2-(2-fluoroethoxy)ethoxy)ethoxy)pyrimidin-2-yl)-[2,2'-bithiazol]-4-yl)-[1,3]dioxolo[4,5-b]pyridine, f_{13}

^{1}H (DMSO-d_6) δ 3.58 – 3.64 (m, 5H), 3.66 – 3.71 (m, 1H), 3.79 – 3.84 (m, 2H), 4.33 – 4.39 (m, 2H), 4.44 – 4.50 (m, 1H), 4.54 – 4.59 (m, 1H), 6.23 (s, 2H), 7.84 (d, *J* = 1.8 Hz, 1H), 8.33 (d, *J* = 1.9 Hz, 1H), 8.34 (s, 1H), 8.55 (s, 1H), 8.69 (s, 2H). ^{13}C (DMSO-d_6) δ 68.27, 68.83, 69.67, 69.82, 69.87, 69.96, 82.44, 83.76, 100.84, 112.29, 116.99, 123.71, 124.43, 136.85, 140.51, 144.31, 151.76, 152.63, 152.66, 154.62, 158.22, 160.59, 160.79.

1.3.11. 4-(benzo[d][1,3]dioxol-5-yl)-4'-(3-(2-(2-(2-fluoroethoxy)ethoxy)ethoxy)-4-methoxyphenyl)-2,2'-bithiazole, f_{14}

^{1}H (DMSO-d_6) δ 3.60 (dt, *J* = 2.0, 6.0 Hz, 2H), 3.61 – 3.67 (m, 3H), 3.67 – 3.72 (m, 1H), 3.77 – 3.84 (m, 5H), 4.17 – 4.23 (m, 2H), 4.43 – 4.49 (m, 1H), 4.52 – 4.59 (m, 1H), 6.10 (s, 2H), 7.01 – 7.06 (m, 1H), 7.08 (d, *J* = 8.4 Hz, 1H), 7.56 – 7.65 (m, 4H), 8.24 (d, *J* = 11.8 Hz, 2H). ^{13}C (DMSO-d_6) δ 55.59, 68.01, 69.05, 69.68, 69.83, 69.90, 69.98, 82.44, 83.76, 101.41, 106.41, 108.73, 111.13, 112.16, 115.30, 115.45, 119.27, 120.30, 126.28, 127.70, 147.61, 147.94, 148.14, 149.48, 155.17, 155.58, 160.15, 160.25.

1.3.12. 4-(benzo[d][1,3]dioxol-5-yl)-4'-(5-methoxypyrimidin-2-yl)-2,2'-bithiazole, f_{15}

^{1}H (DMSO-d_6) δ 3.99 (s, 3H), 6.10 (s, 2H), 7.02 – 7.08 (m, 1H), 7.57 – 7.63 (m, 2H), 8.25 (s, 1H), 8.54 (s, 1H), 8.68 (s, 2H). ^{13}C (DMSO-d_6) δ 56.29, 101.39, 106.42, 108.71, 115.72, 120.32, 123.44, 127.64, 143.85, 147.62, 147.93, 152.30, 152.65, 154.58, 155.20, 160.19, 160.79.

1.3.13. 5-(4'-(5-methoxypyrimidin-2-yl)-[2,2'-bithiazol]-4-yl)-[1,3]dioxolo[4,5-b]pyridine, f_{16}

^{1}H (DMSO-d_6) δ 4.01 (s, 3H), 6.22 (s, 2H), 7.85 (d, *J* = 3.8 Hz, 1H), 8.33 – 8.43 (m, 2H), 8.53 (t, *J* = 2.9 Hz, 1H), 8.68 (t, *J* = 3.0 Hz, 2H). ^{13}C (DMSO-d_6) δ 56.44, 100.03, 111.31, 117.52, 120.71, 123.64, 140.92, 142.11, 142.22, 148.29, 150.03, 151.62, 153.87, 153.89, 160.40, 161.89.

1.3.14. 6-(4'-(4-methoxyphenyl)-[2,2'-bithiazol]-4-yl)-[1,3]dioxolo[4,5-b]pyridine, f_{17}

^{1}H (DMSO-d$_6$) δ 3.82 (s, 3H), 6.23 (s, 2H), 7.03 – 7.09 (m, 2H), 7.84 (d, J = 1.9 Hz, 1H), 7.94 – 7.99 (m, 2H), 8.22 (s, 1H), 8.33 (d, J = 2.6 Hz, 2H). ^{13}C (DMSO-d$_6$) δ 55.28, 100.83, 112.25, 114.34, 115.26, 116.70, 118.19, 124.46, 126.09, 127.61, 136.81, 140.50, 152.62, 155.47, 158.20, 159.60, 160.01, 160.86.

1.3.15. 6-(4'-(3,4-dimethoxyphenyl)-[2,2'-bithiazol]-4-yl)-[1,3]dioxolo[4,5-b]pyridine, f$_{18}$

^{1}H (DMSO-d$_6$) δ 3.71 (s, 1H), 3.83 (s, 4H), 6.23 (s, 3H), 7.07 (d, J = 8.3 Hz, 1H), 7.60 (d, J = 2.0 Hz, 1H), 7.84 (d, J = 1.9 Hz, 1H), 8.28 (s, 1H), 8.32 – 8.37 (m, 3H).

1.3.16. 6-(4'-(2,4-dimethoxyphenyl)-[2,2'-bithiazol]-4-yl)-[1,3]dioxolo[4,5-b]pyridine, f$_{19}$

^{1}H (DMSO-d$_6$) δ 3.84 (s, 3H), 3.95 (s, 3H), 6.23 (s, 2H), 6.67 – 6.74 (m, 2H), 7.83 (d, J = 2.0 Hz, 1H), 8.09 – 8.17 (m, 2H), 8.29 – 8.34 (m, 2H). ^{13}C (DMSO-d$_6$) δ 55.41, 55.73, 98.66, 100.82, 105.61, 112.24, 114.79, 116.54, 118.28, 124.49, 130.21, 136.79, 140.49, 151.52, 152.58, 157.88, 158.17, 158.32, 160.68, 161.06.

1.3.17. 6-(4'-(2,4-bis(2-(2-fluoroethoxy)ethoxy)phenyl)-[2,2'-bithiazol]-4-yl)-[1,3]dioxolo[4,5-b]pyridine, f$_{20}$

^{1}H (DMSO-d$_6$) δ 3.68 – 3.86 (m, 6H), 3.89 – 3.97 (m, 2H), 4.17 – 4.23 (m, 2H), 4.25 – 4.35 (m, 2H), 4.47 – 4.59 (m, 2H), 4.59 – 4.68 (m, 2H), 6.23 (s, 2H), 6.68 – 6.81 (m, 2H), 7.83 (d, J = 1.9 Hz, 1H), 8.14 (d, J = 8.7 Hz, 1H), 8.26 – 8.34 (m, 3H). ^{13}C (DMSO-d$_6$) δ 67.33, 67.51, 68.77, 68.98, 69.63, 69.77, 69.92, 82.47, 83.79, 100.01, 100.81, 106.49, 112.24, 115.08, 116.53, 118.19, 124.49, 130.15, 136.79, 140.49, 151.55, 152.58, 157.00, 158.17, 158.22, 159.73, 161.07.

1.3.18. 6-(4'-(5-(2-(2-(2-iodoethoxy)ethoxy)ethoxy)pyrimidin-2-yl)-[2,2'-bithiazol]-4-yl)-[1,3]dioxolo[4,5-b]pyridine, h$_{13}$

^{1}H (DMSO-d$_6$) δ 3.33 (d, J = 6.4 Hz, 2H), 3.58 – 3.70 (m, 6H), 3.78 – 3.86 (m, 2H), 4.33 – 4.40 (m, 2H), 6.23 (s, 2H), 7.85 (d, J = 1.8 Hz, 1H), 8.31 – 8.37 (m, 2H), 8.55 (s, 1H), 8.70 (d, J = 2.6 Hz, 2H). ^{13}C (DMSO-d$_6$) δ 5.51, 68.28, 68.85, 69.34, 69.96, 71.02, 100.83, 112.28, 116.98, 123.70, 124.42, 136.84, 140.50, 144.32, 151.76, 152.61, 152.65, 154.62, 158.21, 160.57, 160.78.

2. Competition binding assays with [^{3}H]DCVJ

2.1. Alpha-synuclein fibrils

d$_2$

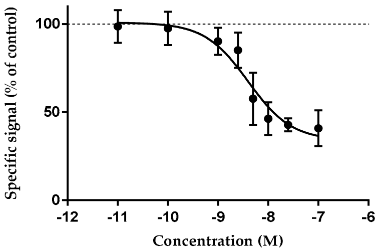

d$_4$

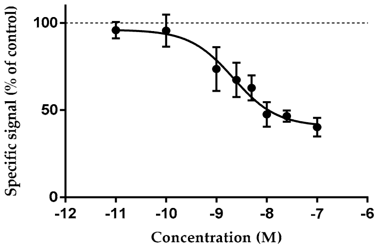

d$_6$

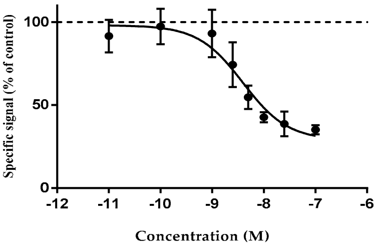

d$_8$

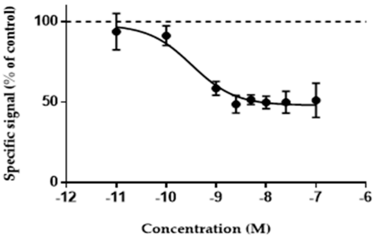

d$_{10}$

f$_4$

f$_5$

f$_6$

f$_7$

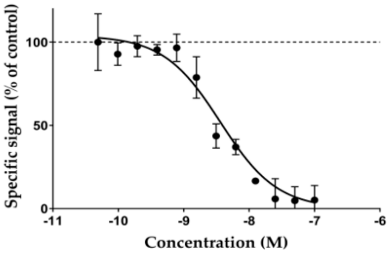

f$_9$

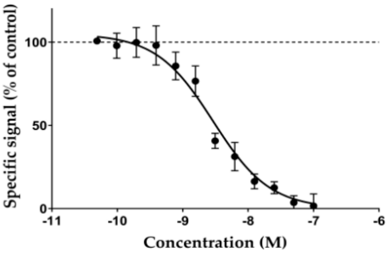

f_{10}

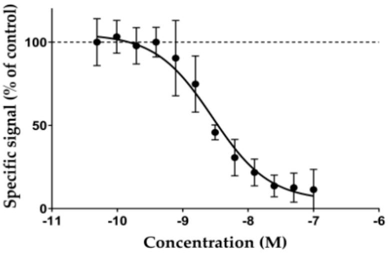

Figure S1. α-Syn displacement binding curves of the some DABTAs against [^{3}H]DCVJ

2.2. Beta-amyloid and tau fibrils

d2

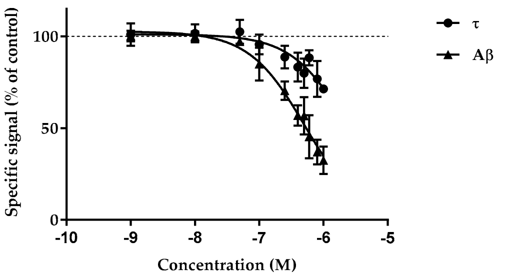

d4

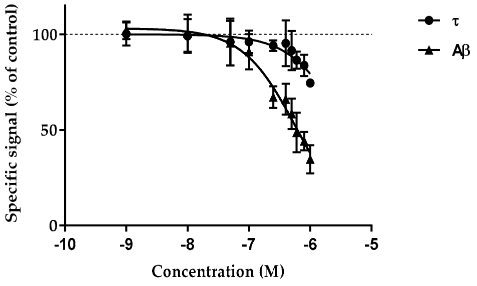

d6

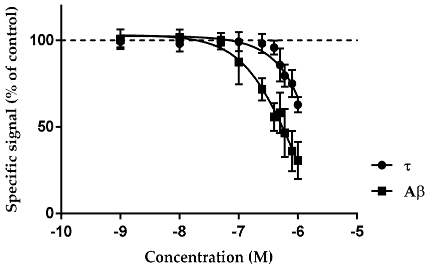

d8

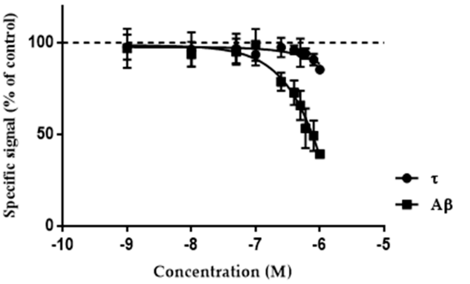

d10

f4

f5

f6

Figure S2. β-amyloid and tau fibrils displacement binding curves of the some DABTAs against [^{3}H]DCVJ

3. Competition binding assays with [^{3}H]PIB

3.1. Alpha-synuclein fibrils

d_2

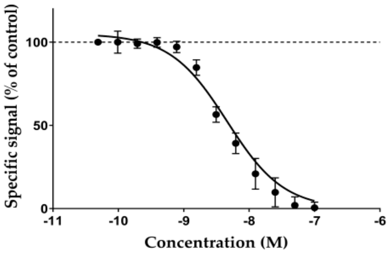

d_4

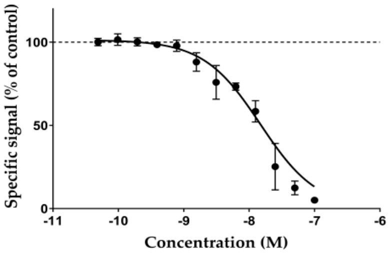

d_6

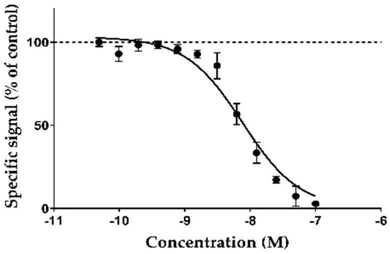

d_8

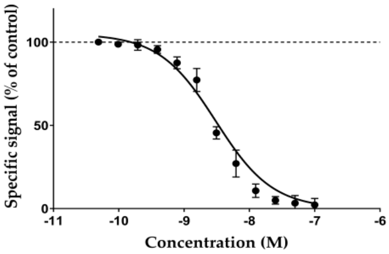

d_{10}

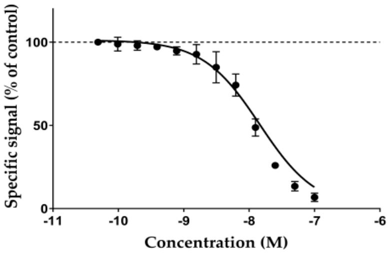

f_4

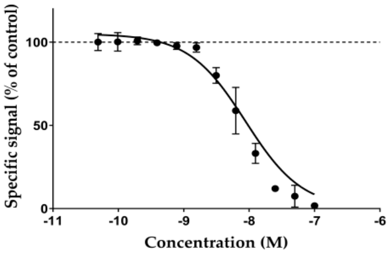

f_5

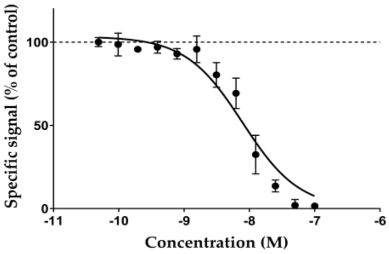

f_6

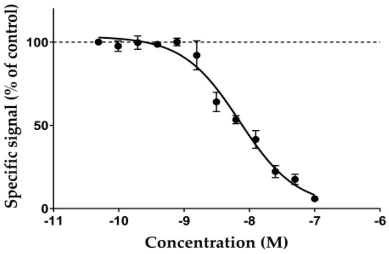

f_7

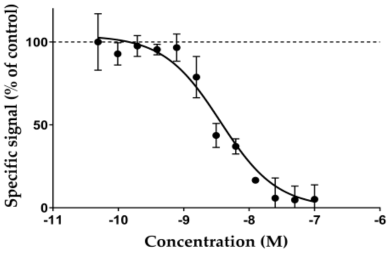

f_8

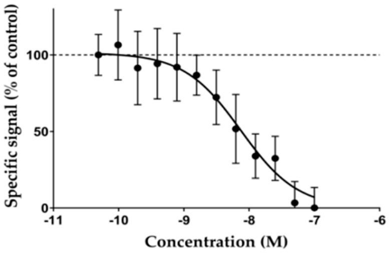

$\mathbf{f_{9}}$

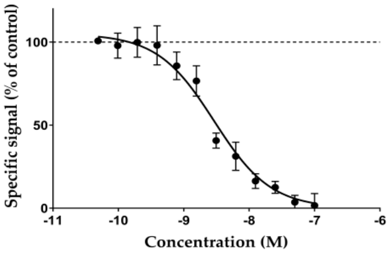

$\mathbf{f_{10}}$

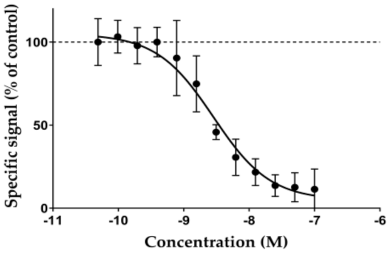

$\mathbf{f_{11}}$

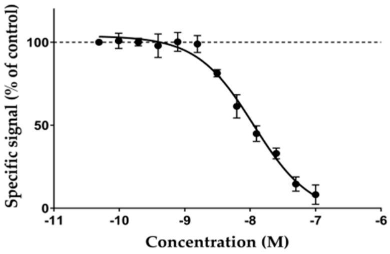

$\mathbf{f_{12}}$

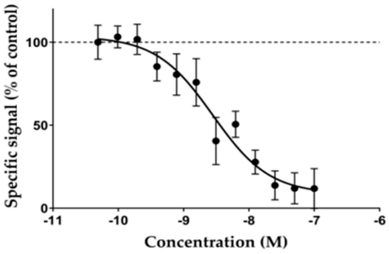

$\mathbf{f_{13}}$

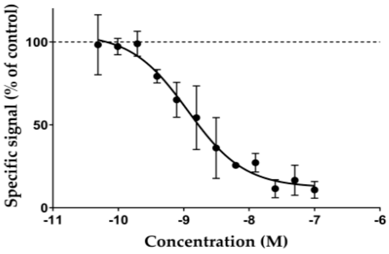

$\mathbf{f_{14}}$

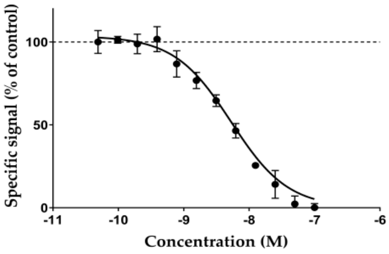

$\mathbf{f_{15}}$

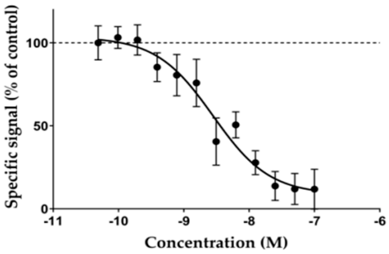

$\mathbf{f_{17}}$

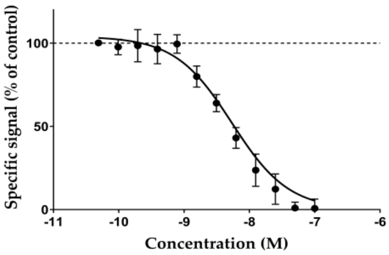

$\mathbf{f_{18}}$

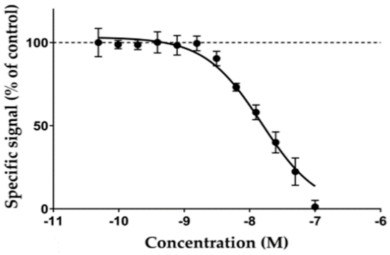

$\mathbf{f_{19}}$

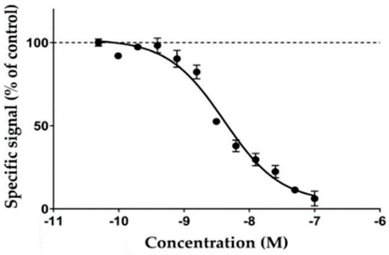

f_{20}

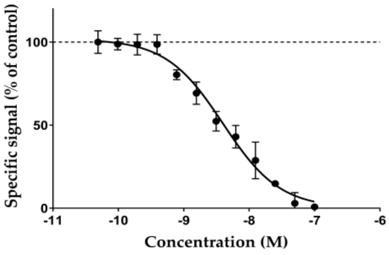

h_{13}

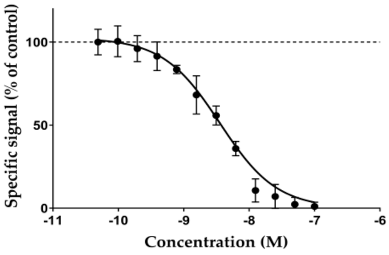

Figure S3. α-Syn displacement binding curves of the all the DABTAs against [^{3}H]PIB

3.2. Beta-amyloid and tau fibrils

d_2

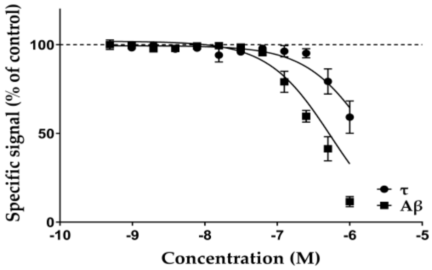

d_4

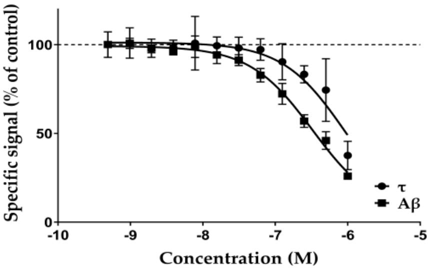

d_6

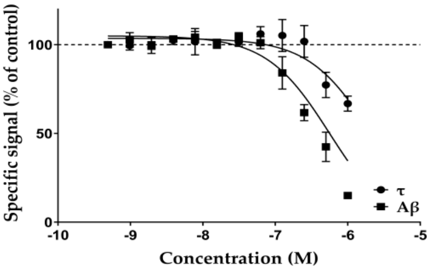

d_8

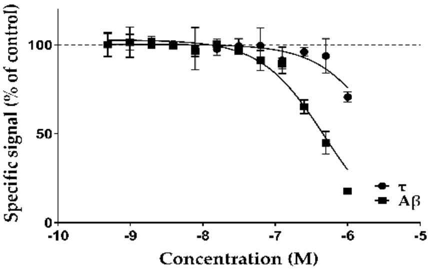

d_{10}

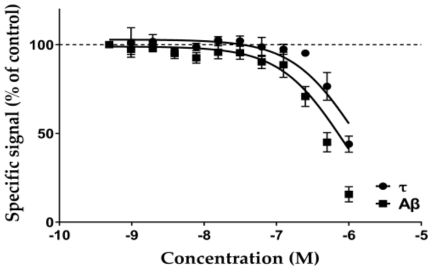

f_4

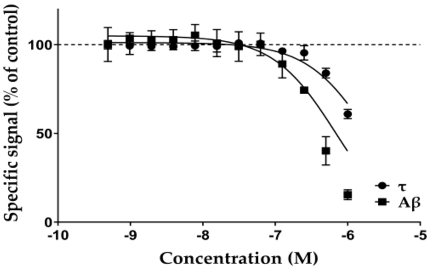

f_5

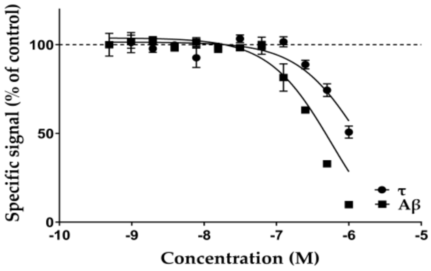

f_6

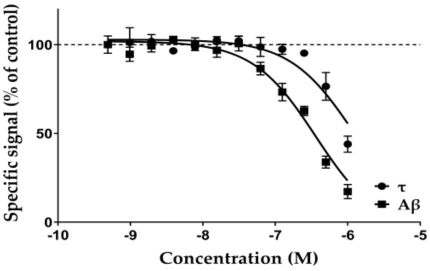

f_7

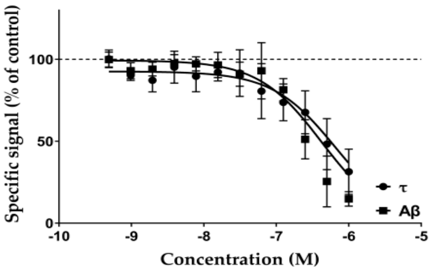

f_9

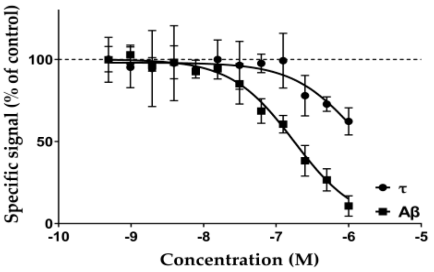

f_{10}

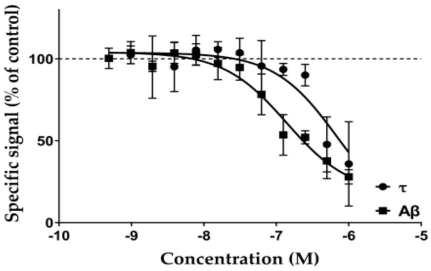

f_{11}

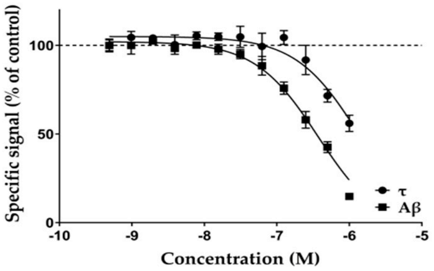

f_{12}

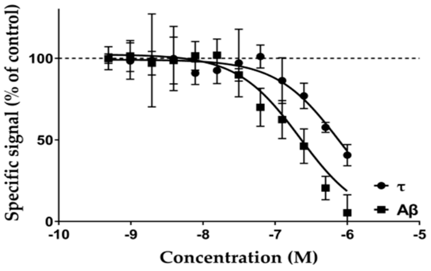

f_{13}

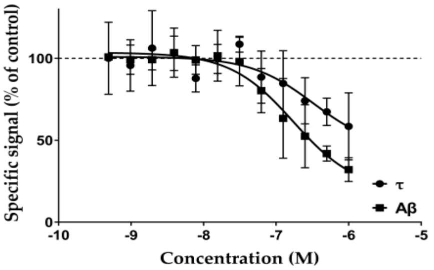

f_{14}

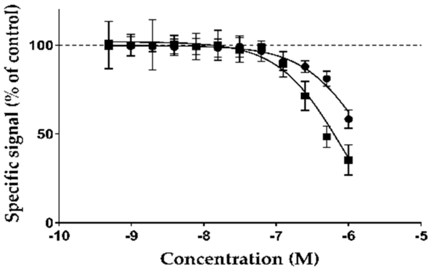

f_{15}

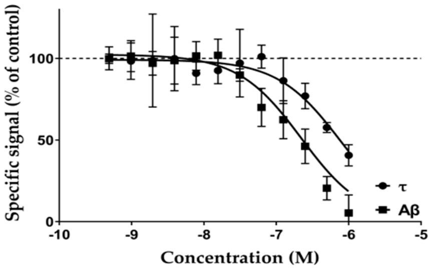

f_{17}

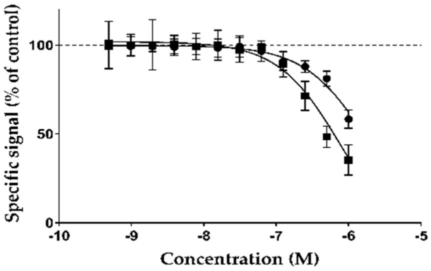

f_{19}

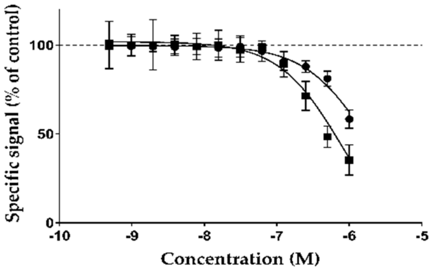

f_{20}

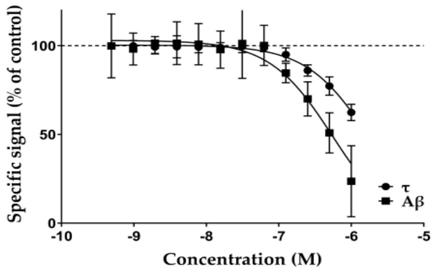

Figure S4. β-amyloid and tau fibrils displacement binding curves all the DABTAs against [^{3}H]PIB

4. In silico studies

	Average over 100-ns				Averaged over top-50 snapshots			
	Site 1	Site 2	Site 3	Site 4	Site 1	Site 2	Site 3	Site 4
f_4	8.3	8.7	2.8	14.1	14.5	14.3	5.2	21.5
f_5	4.3	8.1	2.7	10.9	8.5	17.9	3.5	22.5
f_6	11.3	7.1	3.5	4.6	14.9	13.9	5.5	7.3
f_7	10.4	9.7	2.9	5.2	18.6	14.6	4.3	7.2
f_8	7.0	10.0	4.6	10.4	8.7	16.0	11.1	19.4
f_9	6.1	5.9	3.7	13.5	10.9	8.0	4.8	21.5
f_{10}	5.7	11.5	4.5	11.7	11.9	16.1	5.7	18.8
f_{11}	5.7	10.6	3.5	2.3	13.3	23.1	5.0	3.2
f_{12}	5.4	9.7	4.5	7.7	9.2	16.7	5.2	12.2
f_{13}	7.5	10.0	2.5	8.6	10.9	12.7	3.2	13.7
f_{14}	10.7	7.2	4.9	8.5	16.9	15.8	6.3	12.5
f_{15}	7.5	9.7	4.3	5.5	12.6	15.7	7.7	8.7
f_{16}	6.2	6.1	4.5	15.7	17.3	10.6	5.7	25.7
f_{17}	6.6	4.3	6.3	8.4	15.4	8.6	9.5	11.6
f_{18}	7.1	12.1	2.3	8.9	11.8	17.1	3.9	18.5
f_{19}	5.6	5.7	2.1	4.7	12.4	9.1	3.8	10.7
f_{20}	6.4	7.9	3.8	6.2	8.5	13.1	4.7	8.9
h_{13}	7.5	4.8	5.3	14.8	11.3	11.3	6.1	24.7
d_2	7.4	8.1	2.6	14.0	13.4	11.3	3.4	22.0
d_4	9.9	3.1	3.4	4.7	16.6	7.7	4.6	7.0
d_6	4.6	11.6	7.9	5.1	7.9	23.3	10.2	15.3
d_8	9.0	11.8	3.4	11.6	17.1	21.3	4.2	18.8
d_{10}	7.4	5.0	3.0	20.6	13.1	8.9	4.8	26.9

Figure S5. Root mean square deviations (RMSDs) of ligand (non-hydrogen atoms) in the 23x4 trajectories

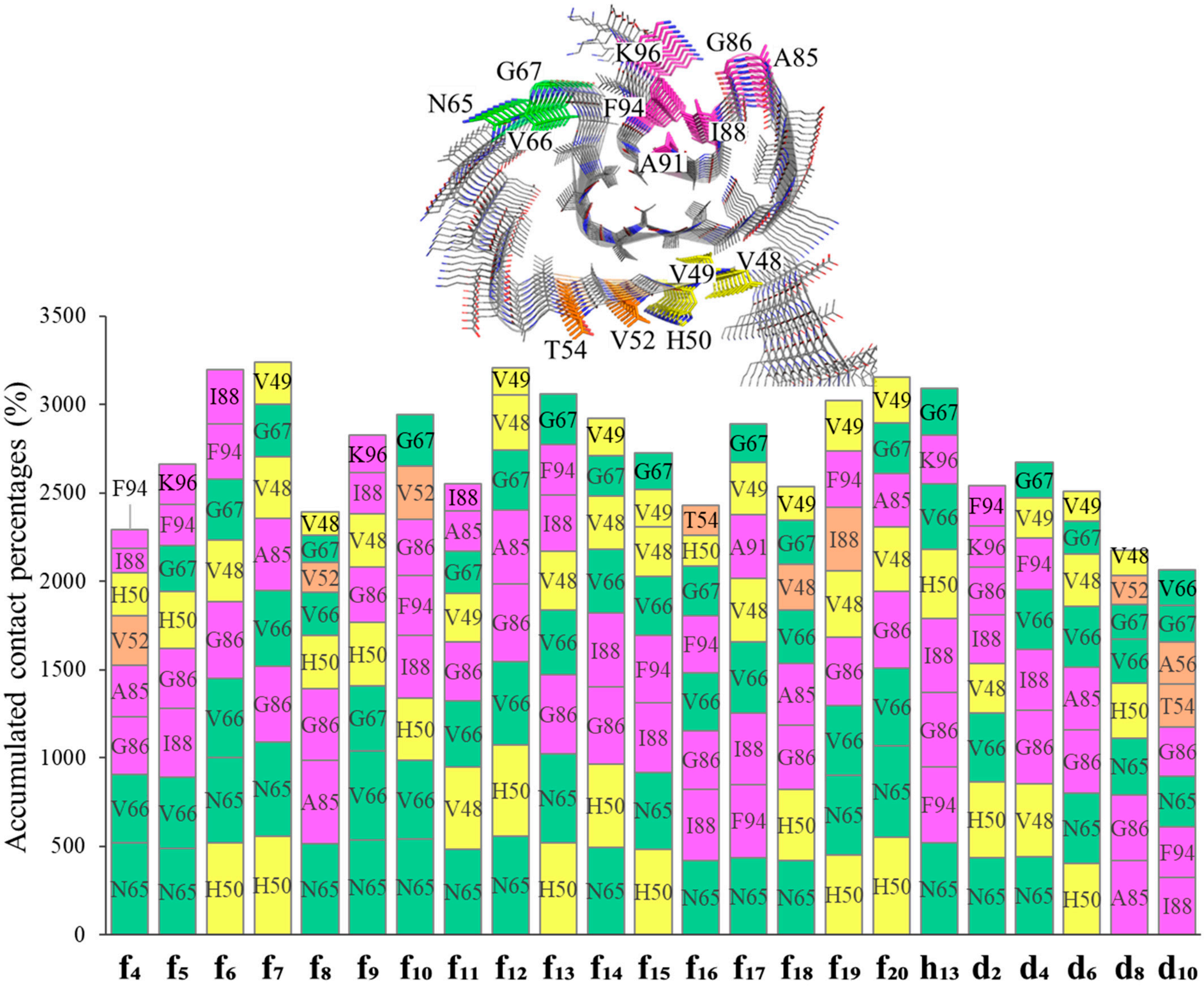

Figure S6. Top-8 most contacted surface residues for each compound on α-Syn protofibril throughout 69 (23 compounds * 3 initial surface sites) 100-ns MD simulations.

4.1. Molecular docking and binding free energy calculations

We adopted the binding sites detected from our previous study with the labels of sites 1-4 [1], in which site-3 is the inner cavity site. The 9-chain protofibril, as it is in the PDB structure, was used and prepared as descripted in previous work [1]. The Glide module in Schrödinger Suite (version 2021-4) was used with the grid center setting to the geometrical center of the residues on the fifth chain of α-Syn protofibril (PDB: 2N0A). To define the grid centers for docking, the geometrical center of the key fifth-chain's residues was adopted for each site, i.e. G67, G68 and K97 for site-1, G86, I88, F94 and K96 for site-2, E61, T72, G73, A53, V55, T59 and E61 for site-3, and K45, V48 and H50 for site-4. The grid size is assigned to 20 Å.

The docked complexes were subjected to the Prime module implemented in Schrödinger Suite (version 2021-4) for MM/GBSA (molecular mechanics, the generalized Born model, and solvent accessibility) binding free energy calculations. The residues within 8 Å of ligand atoms were energy-minimized by the OPLS4 force field in a VSGB (variable dielectric surface generalized Born) continuum solvation model.

4.2. Molecular dynamics simulations

Molecular dynamics (MD) simulations were carried out for the 92 complexes (23 ligands on each of the 4 sites). 100-ns MD simulation was performed for each complex using the Desmond package (version 2021-4) with the similar parameterization of settings to our previous work [2]. Briefly, the OPLS4 force field was used for all the atoms in a solvation box with 10 Å buffering area to the protofibril, which is filled by numbers of sodium/chloride ions (for charge neutralization and a salt concentration of 0.15 M). The Nose-Hoover thermostat and Martyna-Bobias-Klein barostat models were used to maintain the simulations in the NPT ensemble at 300K and 1 atm. Visual Molecular Dynamics (VMD, version 1.9.4a57) and the simulation interaction diagram scripts (implemented in Schrödinger Suite) were used to analyze the trajectories of each system.

References

1. Kuang, G.; Murugan, N.A.; Ågren, H. Mechanistic Insight into the Binding Profile of DCVJ and α-Synuclein Fibril Revealed by Multiscale Simulations. *ACS Chem. Neurosci.* **2019**, *10*, 610–617, doi:10.1021/acschemneuro.8b00465.
2. Li, J.; Kumar, A.; Långström, B.; Nordberg, A.; Ågren, H. Insight into the Binding of First- and Second-Generation PET Tracers to 4R and 3R/4R Tau Protofibrils. *ACS Chem. Neurosci.* **2023**, *14*, 3528–3539, doi:10.1021/acschemneuro.3c00437.
